# Spermatic cord myxoma: A rare case report

**DOI:** 10.1097/MD.0000000000040757

**Published:** 2024-11-29

**Authors:** XuHui Wang, Xinghua Gao, LongYang Zhang

**Affiliations:** a School of Clinical Medicine, Shandong Second Medical University, Weifang, China; b Department of Urology Surgery, Central Hospital Affiliated to Shandong First Medical University, Jinan, China.

**Keywords:** myxoma, spermatic cyst, surgery, case report

## Abstract

**Rationale:**

Spermatic cord myxomas are rare benign tumors, typically characterized by the production of mucopolysaccharides. These tumors are infrequently found in the urinary system, making this case noteworthy.

**Patient concerns:**

A 55-year-old male presented with a painless swelling on the right side of his scrotum, which had persisted for over 3 months. There were no associated symptoms, and the patient had no significant medical history.

**Diagnoses:**

Initial diagnostic workup included physical examination, ultrasonography, and CT scans, which revealed a 2 cm cystic nodule near the right spermatic vein. Postoperative histopathology confirmed the diagnosis of myxoma.

**Interventions:**

The patient underwent laparoscopic high ligation of the left spermatic vein and excision of the right spermatic sheath cyst. The surgery was successful, with the cyst being completely removed.

**Outcomes:**

Postoperative recovery was uneventful, and histopathological examination confirmed the benign nature of the tumor. Follow-up was advised to monitor for any recurrence.

**Lessons:**

Although rare, spermatic cord myxomas should be considered in the differential diagnosis of scrotal masses. Surgical excision is both diagnostic and therapeutic, providing a favorable prognosis with minimal risk of recurrence.

## 1. Introduction

Myxomas are benign tumors that produce large amounts of mucopolysaccharide. Although they are most commonly found in the heart, especially in the left atrium, they can also occur in other parts of the body, such as the skin, bones, and soft tissues.^[1]^ This tumor has a loose cellular structure, is rich in collagen fibers and mucopolysaccharides, and usually presents with a gelatinous or encapsulated texture.^[2]^ Symptoms that may be caused by noncardiac myxoma include localized swelling, pain, or dysfunction, depending on the exact location and size of the tumor. Myxomas located in the urinary system are rare, and only 1 case is reported herein.

## 2. Case report

A 55-year-old male was admitted to the hospital with a self-discovered swelling on the right side of the scrotum that had persisted for more than 3 months. He denied any history of chronic disease. He denied any history of chronic diseases, such as hypertension. He was married at the right age, his spouse and children were healthy, and he denied any family history of hereditary or infectious disease.

**Figure 1.**
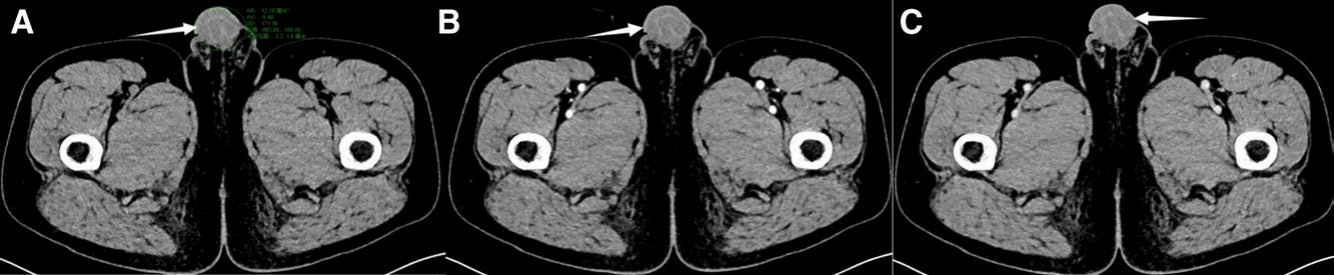
CT showed an obvious mass in the scrotum (A), and the enhanced scans: (B) arterial phase, (C) venous phase showed no enhancement.

On physical examination, the abdomen was soft and nontender, and no pain was noted on percussion in both kidneys, as well as no tenderness in the bilateral ureteric regions. Spermatic varicose veins were palpable in the left and right scrotae. The right scrotum was found to contain an oval-shaped mass, approximately 2 cm in size, without tenderness, soft, and with a negative transillumination test. The skin of the scrotum was neither red nor swollen. Color Doppler ultrasonography showed that the size of the prostate was 3.9 × 3.0 × 3.2 cm, the thickness of the inner gland was 2.1 cm, the peripheral membrane was intact, the echogenicity was uneven, and with patchy strong echoes seen inside. Bilateral seminal vesicles were approximately 1.0 cm thick, and no abnormal echoes were observed. The bilateral testes were normal in size and morphology, with clear borders and homogeneous echoes. The epididymis was normal in size and morphology, and the internal echogenicity was uniform. Multiple cystic nodules were detected on the head of the left epididymis, the largest of which was 0.3 × 0.2 cm in size. Multiple cystic nodules were detected on the head of the right epididymis, the largest of which was 0.7 × 0.5 cm. The left spermatic vein was dilated with the widest internal diameter (2.5 mm) and the reflux test was positive. In contrast, the right spermatic vein was not dilated and the reflux test results were negative. A 2.5 × 1.1 cm cystic nodule was detected in the periphery of the right spermatic vein, with clear borders, multiple reticular-like structures, and multiple blood flow signals. Dual-source enhanced CT (Fig. 1) showed that the bladder was full, and the wall was not thick. The prostate was not large with speckled calcification, and the bilateral seminal vesicles showed no abnormalities. There were no enlarged pelvic lymph nodes or effusions. The right scrotum showed a cystic shadow, approximately 20 × 13 × 16 mm, with clear borders, no obvious enhancement on the enhancement scan, and a few surrounding liquid density shadows. Umbilical ureteral remnants were observed in the anterior wall of the bladder. The remaining relevant examinations (cardiac ultrasound and chest CT) showed no abnormalities.

**Figure 2.**
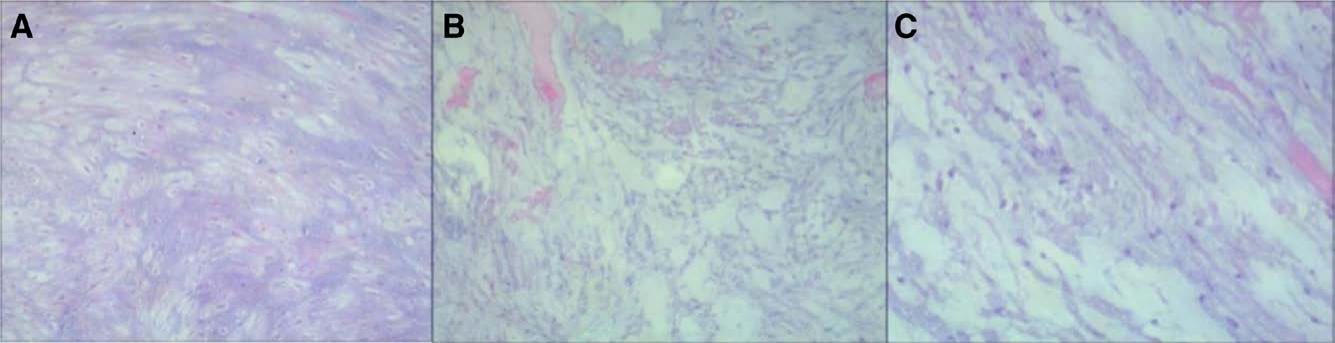
Morphology of the lesion. (A) Low-power view showing large amount of myxoid stroma (HE × 20) and a (B). Marked contrast between the nuclei and the surrounding dense eosinophilic matrix (HE, ×40). (C) High-power showing tumor cells exhibiting minimal pleomorphism (HE, ×100).

Laparoscopic high spermatic vein ligation (left) and spermatic sheath cyst excision (right) were performed on the third day of hospital admission. The total duration of the operation was 77 minutes. During the surgery, the cyst was partially adherent to the spermatic sheath. The cyst was separated from the spematic sheath and peeled off completely, and the surgery proceeded smoothly. Postoperatively, the naked eye examination of the tissue specimen showed a piece of tissue, measuring 2.5 × 1.8 × 1.3 cm. The cut surface showed grayish-white jelly like material with a soft texture. Histopathological examination (Fig. 2) revealed a large amount of myxoid stroma, along with scattered spindle/stellate tumor cells. The tumor cells were mostly arranged around the blood vessels, and a large mucus halo appeared around them, which was in contrast to the surrounding dense eosinophilic stroma. Immunohistochemistry (IHC) showed CK (−), P53 (+, <1%), Ki-67 (+, <1%), S-100 (−), SMA (+), CD34 (vascular+), MyoD1 (−), Desmin (−), CDK4 (+), MDM2 (−), P16 (−), and CD68 (−). The combined morphological and IHC results were consistent with those of spermatic cord myxomas. Therefore a follow-up was recommended.

## 3. Discussion

In 1863, Virchow first introduced the term “myxoma” and in 1948, Stout defined it as a true tumor, marking a deepening of the understanding of these diseases.^[3]^ Myxomas, which are rare soft-tissue tumors, are found primarily in the heart, soft tissues, skin, and bones. They often invade the skeletal muscles of the thighs and buttocks. Myxomas account for up to 50% of benign cardiac tumors, with 75% occurring in the left atrium, 18% in the right atrium, 3% in the right and left ventricles, and 1% in the valves. In contrast to the high recurrence rate of skeletal myxomas, cardiac myxomas have a low recurrence rate, with only 3% of patients recurring because of incomplete resection or conversion to malignancy. Recurrence occurs in a variety of locations, and multiple familial recurrences are not uncommon.^[4]^ In the urinary system, myxomas occur mainly in the kidneys and bladder, and studies have shown that the size of urologic myxomas varies from 4 to 28 cm, and the age of patients ranges from 27 to 82 years. The typical clinical presentation of this tumor is a painless mass with associated compressive symptoms.

Ultrasonography has become the method of choice for diagnosing spermatic cord myxoma because of its ability to demonstrate clear borders and inhomogeneous hyperechoic features of the tumor. CT and enhanced CT often fail to reveal features with high specificity. In contrast, MRI examinations show more specific signal manifestations in diagnosing mucinous neoplasms: low signals on T1WI and high signals on T2WI as well as homogeneous enhancement features during the dynamic phase.^[5]^ Although imaging provides important noninvasive information, the high rate of preoperative misdiagnosis and atypical imaging presentations may still lead to overtreatment.^[6]^

The gold standard for diagnosis is pathological examination, which is performed by analyzing the morphology of tumor cells (e.g., spindle or stellate) and IHC markers6. Patients with spermatic cord myxoma often lack specific clinical symptoms with diagnostic value. Therefore, imaging examination and pathological evaluation are indispensable tools for differential diagnosis, distinguishing it from inguinal hernia and varicocele.^[7]^

In most reported cases, the radiological features of myxoma closely resemble those of malignant tumors, making them susceptible to misdiagnosis as malignant tumors. Therefore, radical resection is the preferred treatment. Pathological evaluation is essential to differentiate mucinous tumors from malignant tumors. If the diagnosis of myxoma can be confirmed preoperatively, tumor removal is adequate not only for diagnosis but also for treatment, and the overall prognosis of the disease is good. After surgical resection, patients with myxomas in the urinary tract do not exhibit invasion, recurrence, or metastasis, further confirming that surgical treatment offers the best prognosis for urological mucinous tumors.^[7]^

Due to its rarity and diagnostic challenges, there are no standard guidelines for the diagnosis or treatment of spermatic cord myxomas. Therefore, the differential diagnosis of other spermatic cord diseases should be considered. Given the risk of relapse after treatment and diagnostic challenges, improving diagnostic accuracy and controlling relapse rates are key to disease management. Future research should focus on the development of accurate diagnostic techniques and effective therapeutic regimens to radically improve patient outcomes and quality of life.

## Acknowledgments

Special thanks to all medical staff of the Department of Urology.

## Author contributions

**Writing – original draft:** XuHui Wang, Xinghua Gao.

**Writing – review & editing:** LongYang Zhang.
